# Association of alkaline phosphatase and segmented neutrophils count with low appendicular lean mass in middle-aged women: Evidence from an American cross-sectional survey

**DOI:** 10.1016/j.tjfa.2026.100213

**Published:** 2026-10-13

**Authors:** Shanlan Yang, Jiaofeng Wang, Dayoung Kim, Han Xu, Sheng Zhou, Fan Yang, Zhijun Bao

**Affiliations:** a Department of Gerontology, Huadong Hospital, Fudan University, Shanghai, PR China; b Shanghai Key Laboratory of Clinical Geriatric Medicine, Huadong Hospital, Fudan University, Shanghai, PR China; c Research Center on Aging and Medicine, Huadong Hospital, Fudan University, Shanghai, PR China; d Shanghai Institute of Geriatrics and Gerontology, Huadong Hospital, Fudan University, Shanghai, PR China.; e Shanghai Institute of Infectious Disease and Biosecurity, Fudan University, Shanghai, PR China.

**Keywords:** Appendicular lean mass, Alkaline phosphatase, Segmented neutrophils count, Machine learning, Middle-aged women

## Abstract

**Objectives:**

Sarcopenia is associated with an increased risk of frailty, falls, and mortality. Women exhibit distinct characteristics in appendicular lean mass and metabolism compared with men, and hormonal changes may further accelerate appendicular lean mass. This study aimed to identify key blood biomarkers associated with appendicular lean muscle mass index adjusted by body mass index (ALMBMI) in women and to explore their potential relationship with chronic inflammation.

**Methods:**

Based on NHANES 2015-2018 data from middle-aged women, the ALMBMI was calculated. A multimodal machine learning framework was constructed using elastic net logistic regression and generalized linear models (GLMs) to screen core variables affecting ALMBMI. Weighted quantile sum (WQS) regression and Bayesian kernel machine regression (BKMR) jointly determined variable weights and effect directions, and restricted cubic spline (RCS) models characterized dose-response curves to identify key blood indicators and their magnitudes of change associated with low appendicular lean mass risk.

**Results:**

A total of 1,082 participants were included in this study. Following variable screening, 12 variables were identified as significantly associated with ALMBMI. WQS regression indicated that segmented neutrophil count (weight: 0.25) and alkaline phosphatase (ALP) (weight: 0.22) were the predominant factors associated with low ALMBMI. BKMR analysis, which assesses all variables jointly, identified segmented neutrophil count (36.0%), and ALP (27.6%) as the core variables. RCS analysis revealed that segmented neutrophil count and alkaline phosphatase showed an L-shaped trend with ALMBMI, with inflection points at 4.00 (1000 cells/μL) and 76.00 IU/L, respectively. In each case, the curves plateaued to the right of the inflection point. Relative to participants classified as having low ALMBMI according to FNIH criteria, those with normal ALMBMI exhibited reductions of 16.01% in ALP and 11.17% in segmented neutrophil count, respectively.

**Conclusion:**

Among middle-aged women, higher segmented neutrophil count and ALP levels were identified as positive correlates of low ALMBMI, with both biomarkers demonstrating threshold phenomena. Such hematological alterations are consistent with chronic low-grade inflammation, underscoring their potential utility in early sarcopenia screening and prevention.

## Highlights

• Alkaline phosphatase and segmented neutrophil count are associated with low appendicular lean mass in middle-aged women.
• Both alkaline phosphatase and segmented neutrophil count exhibit L-shaped nonlinear associations with ALMBMI, with threshold effects.
• Routine blood biomarkers may serve as accessible indicators for screening low appendicular lean mass in middle-aged women.

## 1 Introduction

Sarcopenia[1] is a progressive and generalized skeletal muscle disorder characterized by accelerated loss of muscle strength, mass, and function, seriously contributes to physical disability, increased mortality, and substantial global healthcare expenditures[2]. Furthermore, as skeletal muscle serves as the main regulator of systemic metabolism, sarcopenia is associated with numerous age-related diseases, including diabetes, hypertension, dyslipidemia, osteoporosis, respiratory diseases, cachexia, immunosuppression, and cognitive impairment[3].

Chronic low-grade inflammation (inflammaging) is a hallmark of biological aging[4]. In a chronic inflammatory microenvironment, persistent pro-inflammatory cytokines release disrupts the delicate balance between muscle protein synthesis and degradation, accelerating skeletal muscle atrophy, strength loss, and functional decline, and ultimately leading to sarcopenia[5]. Aging skeletal muscle compromises organismal inflammatory defenses, fosters a pro-inflammatory microenvironment that impairs muscle regeneration, while promoting senescent cells accumulation, culminating in exacerbated atrophy[6^-^7]. Furthermore, it is evidenced that inflammatory biomarkers, particularly serum interleukin-6 and tumor necrosis factor-α levels regulate the occurrence and progression of sarcopenia[8].

Recently, the use of machine learning models for sarcopenia prediction has emerged as a growing trend. For instance, Bo et al. applied an XGBoost model based on ophthalmic examinations and identified that reduced levator function, cataracts, age-related macular degeneration, and blepharophimosis are associated with an increased risk of sarcopenia[9]. Similarly, He et al. used a broad-and-deep model and found that an elevated ratio of alanine aminotransferase to aspartate aminotransferase is a characteristic indicator of sarcopenia in older adults[10]. Nevertheless, applying traditional machine learning models for sarcopenia risk prediction and factor identification has three key limitations. First, most existing studies have focused primarily on the elderly population. However, recent evidence suggests that accelerated declines in muscle strength, mass, and function begin as early as middle age[11]. Second, existing evidence indicates that the rate of decline in muscle mass and muscle function is higher in middle-aged women than in middle-aged men, which may be associated with the sharp decline in estrogen following menopause in women[12]. Third, sarcopenia is a relatively rare event among middle-aged and older adults. Traditional machine learning models trained on such imbalanced data tend to learn decision boundaries biased toward the majority class (i.e., non-sarcopenic individuals), resulting in poor sensitivity in identifying actual sarcopenia cases. Finally, existing studies largely rely on single types or specific systems of indicators, making it difficult for individuals with or at risk of sarcopenia to detect early signs of the disease in their daily lives.

Therefore, the focus of this study is to use comprehensive and easily accessible blood indicators to identify key blood markers associated with low appendicular lean mass in middle-aged women, while mitigating the impact of class imbalance in model fitting. To this end, we converted the outcome variable from a binary classification to a continuous measurement, using the body mass index-adjusted appendicular skeletal muscle mass index (ALMBMI) as the dependent variable, thereby reducing the inherent imbalance in the data. Routine blood and biochemical indicators reflecting the health status of multiple physiological systems during physical examinations were included in the study. Given the high dimensionality and potential multicollinearity among these blood markers, we applied a machine learning framework that integrates multiple mathematical models. Elastic net linear regression and generalized linear regression were used to screen and identify the core variables influencing ALMBMI and their directions. Weighted quantile sum (WQS) regression and Bayesian kernel machine regression (BKMR) were jointly applied to estimate the weight distribution of the variables, and restricted cubic splines (RCS) were used to describe their dose-response curves. This multi-model approach simplifies the complexity of multidimensional physical examination data and robustly reveals the associations between these variables and low appendicular lean mass, providing potential screening indicators for low appendicular lean mass in the middle-aged female population. Furthermore, this approach visualizes the inflammatory status associated with low appendicular lean mass in the middle-aged female population, thereby supporting early multi-system monitoring and intervention for high-risk groups while maximizing the utilization of healthcare resources.

## 2 Methods

### 2.1 Participants

The National Health and Nutrition Examination Survey (NHANES) is a biannual survey conducted by the National Center for Health Statistics (NCHS). The NCHS Ethics Review Board approved the study and all participants provided written informed consent. Detailed NHANES study information and data are available online (www.cdc.gov/nchs/nhanes/). Using the NHNAES 2015-2018 database, women individuals aged 45-59 years who had complete blood count, standard biochemistry, high-sensitivity C-reactive protein (hs-CRP), body mass index (BMI), and dual-energy X-ray absorptiometry (DXA) measurements were included. Participants under 45 years of age (n=6,260) or with missing data on weight (n=35), DXA (n=2,368), or standard biochemistry (n=51) were excluded. Final sample comprised 1,082 participants.

### 2.2 Blood-based indicators

Three types of data from the NHANES database were selected as blood-based indicators: (1) complete blood count (CBC), a routine and universal blood test used to assess overall health status and detect various conditions; (2) standard biochemical panel, used to evaluate the functional status of internal organs, metabolic activity, and electrolyte balance by measuring the concentrations of various chemical components in the blood; and (3) high-sensitivity C-reactive protein (Hs-CRP), a conventional and important clinical biomarker primarily used for monitoring inflammatory levels and assessing disease risk.

### 2.3 Low appendicular lean mass and sarcopenia

Appendicular lean muscle mass (ALM) was calculated by summing the muscle mass of all four limbs using DXA. According to the guidelines formulated by the National Institutes of Health Foundation of the United States (FNIH), sarcopenia (low appendicular lean mass) was defined as ALM normalized to BMI (ALMBMI), with cutoff values of less than 0.512 for females[13].

### 2.4 Potential confounding variables

Demographic data were obtained from the participants by face-to-face interviews, including age, gender, BMI, education level, marital status, family poverty income ratio (PIR), status of alcohol consumption, smoking, and exercise. The BMI was defined as body weight (kg)/height^2^ (m^2^). Drinking and smoking status was assessed by the participants' response to the question, “During the past 12 months, how often did you consume any type of alcoholic beverage?” Smoking status was based on the question, “Do you now smoke cigarettes?” Exercise status was calculated as the combined total of moderate and vigorous physical activity. Information on kidney disease, hypertension, high cholesterol, gastrointestinal diseases and diabetes was included in the analysis, and disease prevalence was determined from participants’ self-reported diagnoses by medical professionals.

### 2.5 A multi‑model machine learning framework integrating diverse mathematical approaches

A total of 55 indicators were included in this study, with certain correlations among them. Therefore, prior to model building, we employed a combined filter and embedded method to screen for indicators significantly associated with changes in ALMBMI. For filter-based feature selection, indicators with inter-variable correlations exceeding 0.7 were removed. For embedded selection, we used an elastic net linear regression model[14^-^15], which simultaneously performs feature filtering and handles multicollinearity by adding L1 (Lasso) and L2 (Ridge) penalties to the linear regression loss function. The optimal mixing parameter α and regularization parameter λ were determined through a two‑stage grid search combined with 10‑fold cross‑validation, minimizing the mean squared error (MSE). Considering the simplicity of the indicators, λ₁SE (the largest λ within one standard error of the minimum MSE) was selected to obtain the final model. The final dataset consisted of all non-zero feature variables, which were subsequently incorporated into a generalized linear regression model to estimate the sign and magnitude of the regression coefficients (β) for each variable on ALMBMI.

The variables retained after initial screening were further evaluated for multiple effects using the WQS model and the BKMR model. The WQS model was originally developed to identify the effects of high-dimensional mixtures of environmental chemicals on an outcome[16]. It transforms multi-dimensional continuous variables into weighted quantile variables, accounting for directional effects while estimating variable weights. Since WQS regression requires the included variables to have consistent direction of association, variables with positive and negative effects on ALMBMI were separated into two independent matrices for separate analyses. For each matrix, the data were randomly split into training and validation sets at a 7:3 ratio; the process was repeated 20 times, and the weight estimates from each iteration were averaged to obtain the final weight for each variable. In the primary analysis, variables were categorized into quartiles (q = 4), while sensitivity analysis was performed with q = 5.

BKMR is a non‑parametric statistical model widely used by environmental epidemiologists to analyze associations between exposure mixtures and health outcomes. Owing to its flexibility and interpretability, it has recently been extended to social epidemiological studies involving continuous variables[17^-^18]. Before constructing the BKMR model, all continuous variables were standardized to eliminate the effects of different units in this study. Model estimation was conducted using Markov Chain Monte Carlo (MCMC) sampling with 20,000 iterations, a burn‑in period of 10,000, and a thinning interval of 5. The variables screened from the WQS analyses were collectively incorporated into the BKMR model to obtain the overall integrated weights. The BKMR model allowed us to calculate variable weights, overall effects, single-risk effects, and variable importance. To verify the reliability of the MCMC sampling and model convergence, we assessed: (1) the trace plots of the global smoothing parameter λ and the weight parameters θm across the 20,000 iterations to ensure stable fluctuations without long‑term trends or periodic patterns; and (2) the autocorrelation coefficients of each variable at lags 0–30, which showed rapid decay toward zero with increasing lag order, indicating good sampling independence.

After screening for variables highly correlated with ALMBMI, we used RCS models to visualize the relationships between these variables and ALMBMI and to determine whether non‑linear trends existed. RCS models are a powerful tool for visualizing dose‑response relationships between continuous indicators and outcomes. This approach partitions the range of a continuous variable into several intervals, fits ordinary least squares linear regression within each interval, and ensures smoothness by constraining the first and second derivatives to be continuous at all knots. For each RCS model, 3, 4, and 5 knots were tested; the optimal number of knots for each variable was selected based on the Akaike information criterion for final fitting. Overall and non‑linear effects of each variable were assessed using F-tests from analysis of variance. To avoid unstable extrapolation at both ends of the data range, linearity was forced at the boundaries.

### 2.6 Statistical analyses

All analyses were performed using R software (version R 4.4.3, developed by the Foundation for Statistical Computing, Vienna, Austria). Continuous variables are presented as mean ± standard deviation, and categorical variables as proportions. A *P*-value <0.05 was considered statistically significant. In studies based on the NHANES database, sample weighting was not applied, as adjustments for demographic factors were performed[19].

## 3 Results

### 3.1 Baseline characteristics

Based on the NHANES database from 2015 to 2018, a total of 1,082 participants who met the study criteria were included, comprising 151 individuals classified as having low ALMBMI (13.96%) and 931 with normal ALMBMI (86.04%) according to the FNIH criteria. The overall mean ALMBMI was 0.61 ± 0.09. Table 1 shows the comparison between participants classified as having low ALMBMI and those with normal ALMBMI. The primary variables assessed in this study were routine blood parameters (21 indicators) and serum biochemical parameters (24 indicators), as presented in Table 2, Table 3. To address the class imbalance issue inherent in machine learning prediction of binary outcomes, the continuous variable ALMBMI was employed to evaluate muscle health.

**Table 1:** Demographic characteristics and comparisons of participants by ALMBMI status.

Variables		Total	Low ALMBMI	Normal ALMBMI
		(n=1,082)	(n=151)	(n=931)
ALMBMI		0.61±0.09	0.47±0.03	0.63±0.08
Age (years)	45∼50	342 (31.61%)	38 (25.17%)	304 (32.65%)
	51∼55	364 (33.64%)	51 (33.77%)	313 (33.62%)
	56∼60	376 (34.75%)	62 (41.06%)	314 (33.73%)
Education level	Less than 9th grade	205 (18.95%)	54 (35.76%)	151 (16.22%)
	9-11th grade	249 (23.01%)	40 (26.49%)	209 (22.45%)
	High school graduate	336 (31.05%)	37 (24.50%)	299 (32.12%)
	College	292 (26.99%)	20 (13.25%)	272 (29.22%)
Marital status	Married	663 (61.28%)	97 (64.24%)	566 (60.79%)
	Never married	419 (38.72%)	54 (35.76%)	365 (39.21%)
Kidney disease	Yes	32 (2.96%)	8 (5.30%)	24 (2.58%)
	No	1,050 (97.04%)	143 (94.70%)	907 (97.42%)
Hypertension	Yes	420 (38.82%)	55 (36.42%)	365 (39.21%)
	No	662 (61.18%)	96 (63.58%)	566 (60.79%)
High cholesterol	Yes	402 (37.15%)	58 (38.41%)	344 (36.95%)
	No	680 (62.85%)	93 (61.59%)	587 (63.05%)
Gastrointestinal diseases	Yes	92 (8.50%)	24 (15.89%)	68 (7.30%)
	No	990 (91.50%)	127 (84.11%)	863 (92.70%)
Diabetes	Yes	156 (14.42%)	40 (26.49%)	116 (12.46%)
	No	926 (85.58%)	111 (73.51%)	815 (87.54%)
Cigarette	Every day	167 (15.43%)	17 (11.26%)	150 (16.11%)
	Some days	132 (12.20%)	109 (72.19%)	23 (2.47%)
	Never used	783 (72.37%)	25 (16.56%)	758 (81.42%)
Continued Table 1 Demographic characteristics and comparisons of participants				

Variables		Total	Low ALMBMI	Normal ALMBMI
		(n=1,082)	(n=151)	(n=931)
Alcohol	Never used	184 (17.01%)	35 (23.18%)	149 (16.00%)
	Every day	223 (20.61%)	25 (16.56%)	198 (21.27%)
	Every week	204 (18.85%)	65 (43.05%)	139 (14.93%)
	Every mouth	126 (11.65%)	9 (5.96%)	117 (12.57%)
	Every year	345 (31.89%)	17 (11.26%)	328 (35.23%)
Exercise	Yes	457 (42.24%)	39 (25.83%)	418 (44.90%)
	No	625 (57.76)	112 (74.20%)	513 (55.10%)
PIR		2.72±1.68	2.16±1.51	2.81±1.69

**Note:** Data are presented as mean ± SD for continuous variables and as *n* (%) for categorical variables. ALMBMI, appendicular lean mass index adjusted by body mass index; PIR, poverty income ratio. ALMBMI < 0.512 was defined as low ALMBMI according to the FNIH criteria.

**Table 2:** Blood routine test and comparisons of the participants by ALMBMI status.

Variables	Total	Low ALMBMI	Normal ALMBMI
	(n=1,082)	(n=151)	(n=931)
Hs-CRP (mg/L)	5.51±11.4	7.79±11.22	5.14±11.40
White blood cell count (1000 cells/uL)	7.33±2.58	7.85±2.05	7.24±2.65
Lymphocyte percentage (%)	32.92±8.90	31.86±7.62	33.09±9.09
Monocyte percentage (%)	7.53±2.02	7.32±1.59	7.57±2.08
Segmented neutrophil percentage (%)	56.17±9.37	57.76±7.97	55.91±9.56
Eosinophil percentage (%)	2.70±2.07	2.42±1.71	2.74±2.12
Basophil percentage (%)	0.81±0.34	0.76±0.32	0.82±0.34
Lymphocyte count (1000 cells/uL)	2.36±0.95	2.47±0.79	2.34±0.97
Monocyte count (1000 cells/uL)	0.54±0.20	0.56±0.16	0.53±0.21
Neutrophil count (1000 cells/uL)	4.19±1.83	4.58±1.54	4.12±1.87
Eosinophil count (1000 cells/uL)	0.20±0.17	0.19±0.13	0.20±0.17
Basophil count (1000 cells/uL)	0.06±0.06	0.06±0.05	0.06±0.06
Red blood cell count (1000 cells/uL)	4.60±0.41	4.66±0.35	4.59±0.42
Hemoglobin (g/dL)	13.36±1.27	13.35±1.29	13.36±1.27
Hematocrit (%)	40.23±3.47	40.25±3.42	40.23±3.47
Mean corpuscular volume (fL)	87.75±6.74	86.62±6.51	87.94±6.76
Mean corpuscular hemoglobin (pg)	29.13±2.66	28.73±2.62	29.19±2.67
Mean corpuscular hemoglobin concentration (g/dL)	33.16±1.03	33.13±0.91	33.17±1.05
Red cell distribution width (%)	13.97±1.52	14.19±1.72	13.93±1.49
Platelet count (1000 cells/uL)	259.04±66.62	273.66±73.40	256.67±65.19
Mean platelet volume (fL)	8.35±0.90	8.40±0.98	8.34±0.88

**Note:** Data are presented as mean ± *SD*. Hs-CRP, high-sensitivity C-reactive protein.

**Table 3:** Standard blood biochemistry and comparisons of the participants by ALMBMI status.

Variables	Total	Low ALMBMI	Normal ALMBMI
	(n=1,082)	(n=151)	(n=931)
Alanine aminotransferase (U/L)	22.97±17.06	23.10±11.64	22.95±17.79
Albumin (g/L)	41.11±3.32	40.46±3.18	41.22±3.33
Alkaline phosphatase (IU/L)	79.39±27.09	90.09±28.310	77.66±26.50
Aspartate aminotransferase (U/L)	24.20±27.87	21.99±9.79	24.56±29.77
Bicarbonate (mmol/L)	25.08±2.33	24.91±2.45	25.11±2.31
Blood urea nitrogen (mmol/L)	4.84±1.53	4.90±1.56	4.83±1.52
Chloride (mmol/L)	102.29±2.98	102.31±2.94	102.29±2.99
Creatine phosphokinase (IU/L)	128.89±269.54	91.95±54.43	134.89±289.33
Creatinine (umol/L)	64.91±18.58	58.24±19.56	65.99±18.20
Globulin (g/L)	30.56±4.74	31.62±3.57	30.39±4.89
Gamma-glutamyl transferase (U/L)	31.11±45.86	30.72±27.44	31.18±48.20
Glucose (mmol/L)	5.80±2.43	6.33±3.10	5.71±2.29
Iron (umol/L)	14.05±6.12	13.20±5.86	14.19±6.15
Lactate dehydrogenase (U/L)	145.79±32.19	144.3±31.39	146.04±32.32
Osmolality (mmol/Kg)	278.68±5.20	279.53±5.59	278.55±5.12
Phosphorus (mmol/L)	1.20±0.17	1.19±0.16	1.21±0.17
Potassium (mmol/L)	3.92±0.34	3.93±0.34	3.92±0.34
Sodium (mmol/L)	139.41±2.51	139.58±2.85	139.38±2.45
Total bilirubin (umol/L)	7.52±4.09	7.09±3.26	7.59±4.20
Total calcium (mmol/L)	2.32±0.09	2.31±0.10	2.33±0.09
Total cholesterol (mmol/L)	5.38±1.02	5.31±10	5.39±1.02
Total protein (g/L)	71.67±4.37	72.08±3.39	71.60±4.51
Triglycerides (mmol/L)	1.69±1.12	1.81±0.85	1.67±1.16
Uric acid (umol/L)	286.70±71.67	296.92±87.85	285.04±68.61

**Note:** Data are presented as mean ± *SD*.

### 3.2 Screening of factors associated with low ALMBMI in middle-aged women

A filtering method was first applied to exclude variables with correlation coefficients exceeding 0.7, resulting in the removal of eight variables (including white blood cell count, hemoglobin, and mean corpuscular hemoglobin) (Supplementary Figure 1). The remaining continuous variables were standardized, and categorical variables were dummy-coded, followed by screening using elastic net linear regression (an embedded method). The model was fitted using 10-fold cross-validation, yielding an optimal elastic net parameter α of 0.8 and a regularization parameter λ of 0.0114 (Supplementary Figure 2). Ultimately, 12 variables were identified as significantly associated with ALMBMI, including demographic information, routine blood markers, and serum biochemical indicators. A regression coefficient (β) > 0 indicates that as the variable increases, ALMBMI increases, representing a factor negatively associated with low ALMBMI; β < 0 indicates that as the variable increases, ALMBMI decreases, representing a factor positively associated with low ALMBMI. Excluding demographic variables, five factors positively associated with low ALMBMI and two factors negatively associated with low ALMBMI were identified in women (Table 4).

**Table 4:** Factors associated with low ALMBMI in middle-aged women identified by elastic net linear regression.

**Variable**	***β***	***SE***	***P***
**Diabetes**			
No	Ref		
Yes	-0.017	0.0069	0.0142
**Smoke**			
Every Day	Ref		
Some Days	-0.1103	0.0092	<0.0001
Not Used	-0.0094	0.007	0.1799
**Uric acid**	-0.0002	0	<0.0001
**ALP**	-0.0003	0.0001	0.0005
**Segmented neutrophils num**	-0.004	0.0014	0.0047
**Platelet count**	-0.0001	0	0.0062
**Triglycerides**	-0.002	0.0022	0.3782
**Education**			
Less Than 9th Grade	Ref		
9-11th Grade	0.0129	0.0074	0.084
High School Graduate	0.0158	0.0073	0.0297
College	0.0239	0.008	0.0029
**Exercise**			
No	Ref		
Yes	0.0168	0.005	0.0008
**PIR**	0.0041	0.0016	0.0109
**CPK**	<0.0001	<0.0001	0.0001
**Creatinine**	0.0009	0.0001	<0.0001

Note: Variables with regression coefficient (*β*) < 0 indicate that as the variable increases, ALMBMI decreases, representing factors positively associated with low ALMBMI. Variables with *β* > 0 indicate that as the variable increases, ALMBMI increases, representing factors negatively associated with low ALMBMI. *SE*, Standard Error; ALP, Alkaline Phosphatase; PIR, Poverty Income Ratio; CPK, Creatine Phosphokinase.

### 3.3 Direction and weighted of blood indicators on ALMBMI in the WQS model

In this study, variables with positive and negative regression coefficients were analyzed as two separate composite indices in the WQS model. Variables were categorized into quartiles (q = 4) to calculate relative importance, with full adjustment for confounders and sensitivity analysis performed using q = 5. These direction-specific weights represent the relative contribution of each variable within its respective matrix. The WQS analysis results are shown in Fig. 1. Among females, the analysis of variables positively associated with low ALMBMI revealed that segmented neutrophil count (weight: 0.25) and ALP (weight: 0.22) together accounted for 47% of the total weight. Triglycerides, uric acid, and platelet count had weights of 0.19, 0.18, and 0.15, respectively. The analysis of variables negatively associated with low ALMBMI indicated that creatinine had a dominant weight of 0.82, whereas creatine phosphokinase had a weight of only 0.18. The results of sensitivity analysis (q=5) were consistent with the main findings.

**Fig. 1:**
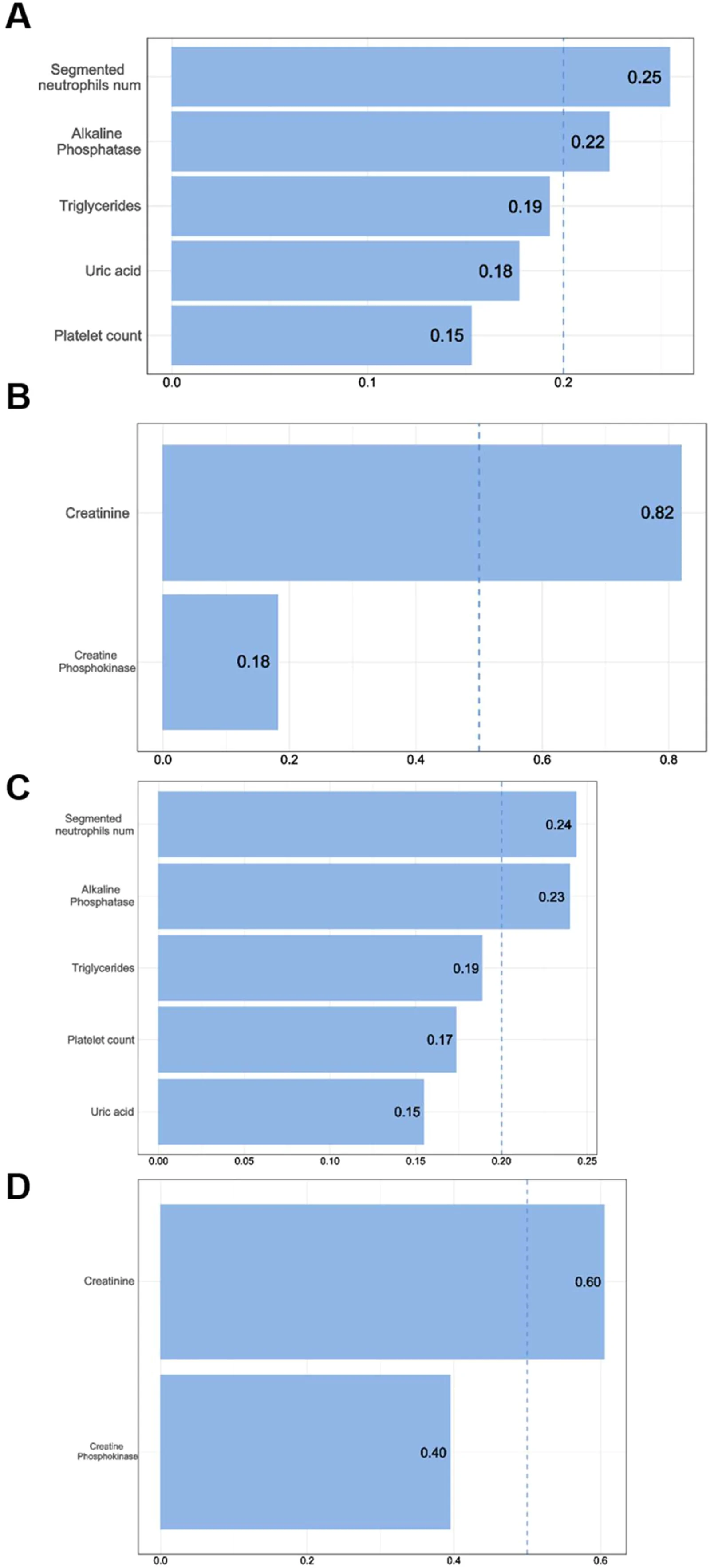
Direction and weight associations of blood indicators with ALMBMI in the WQS model. Note: (A) Variables positively associated with low ALMBMI. (B) Variables negatively associated with low ALMBMI. (C) Variables positively associated with low ALMBMI in sensitivity analysis (Q=5). (D) Variables negatively associated with low ALMBMI in sensitivity analysis (Q=5).

### 3.4 Weights and associations of blood indicators with ALMBMI in the BKMR model

To further determine the contribution of core variables, those with relatively higher contributions in the WQS model were included in the BKMR model for additional analysis, with all analyses fully adjusted for confounders. Unlike the WQS model, which evaluates variables separately by direction, the BKMR model assesses all variables jointly and provides integrated weights that reflect the relative importance of each variable when all are considered simultaneously, accounting for potential interactions. The reliability and convergence of the MCMC sampling were assessed by examining the trace plots of the global smoothing parameter λ and the weight parameters θm across the 20,000 iterations, which showed stable fluctuations without long-term trends or periodic patterns, as well as by analyzing the autocorrelation coefficients of each variable at lags 0-30, which demonstrated rapid decay toward zero with increasing lag order, indicating good sampling independence (Supplementary Fig. 3, Supplementary Table 1). The BKMR results for females are presented in Fig. 2. Variable relative importance analysis showed that creatinine and segmented neutrophil count had relatively higher weights (36.4% and 36.0%, respectively), while alkaline phosphatase had a lower weight (27.6%). These BKMR-derived integrated weights differ from the WQS direction-specific weights because the two models address distinct analytical questions. When the other two indicators were fixed at *P_25_, P_50_*, and *P_75_*, creatinine consistently exhibited a positive effect, whereas segmented neutrophil count and alkaline phosphatase showed consistently negative effects. With other indicators fixed at their median levels, the exposure-response relationship curves for each individual variable further confirmed the negative effects of segmented neutrophil count and alkaline phosphatase and the positive effect of creatinine.

**Fig. 2:**
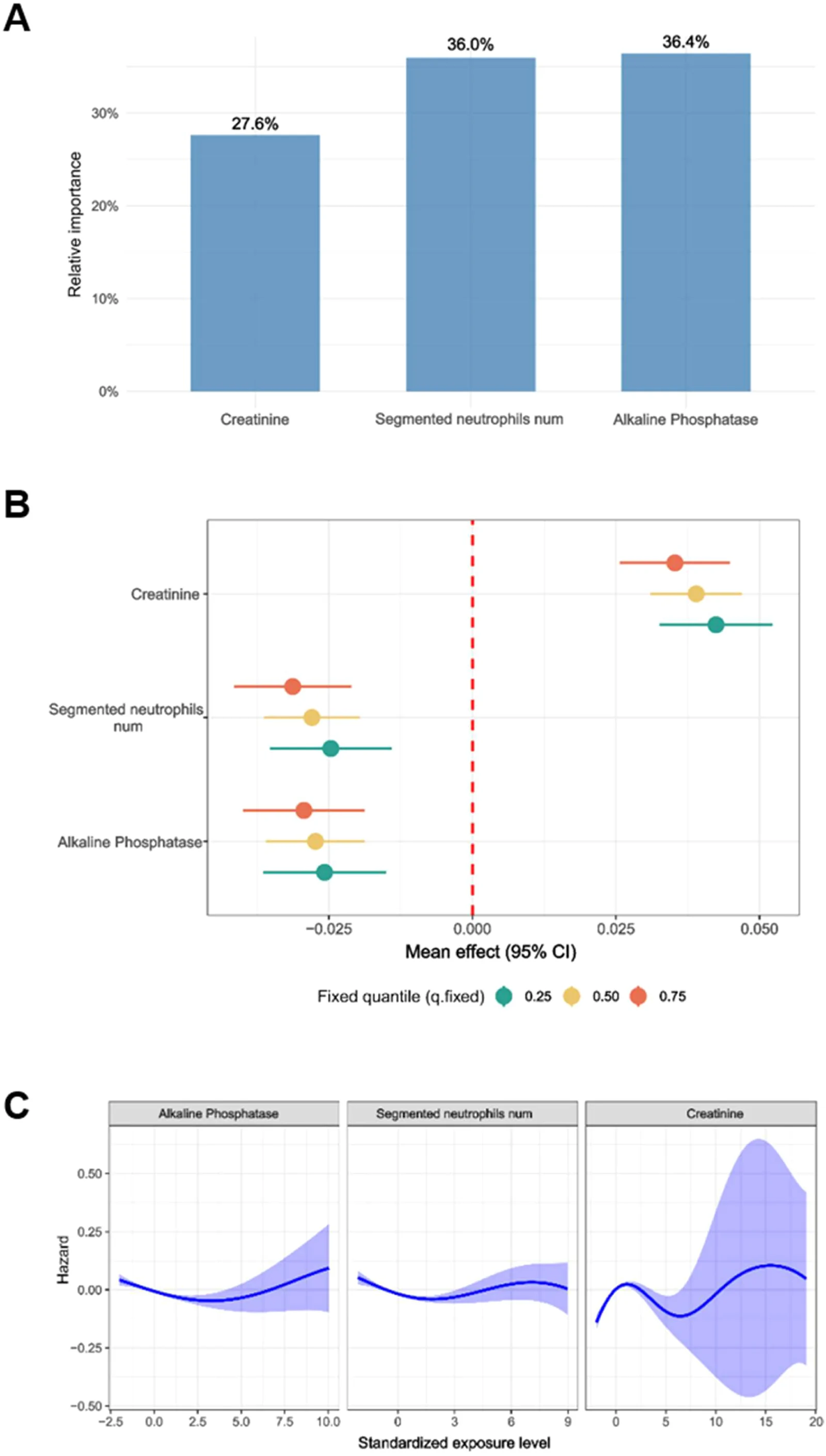
Variable importance and single-exposure associations in the BKMR model. **Note:** (A) Relative importance of variables. (B) Single-exposure association plots. (C) Curves of the corresponding relationship for single exposure.

### 3.5 Regression and inflection point associations of blood indicators with ALMBMI in the RCS model

To characterize the dose-response relationships between multidimensional blood variables and ALMBMI, further analysis was conducted using the RCS method after adjusting for confounders. In females, creatinine, segmented neutrophil count, and alkaline phosphatase exhibited significant nonlinear associations with ALMBMI (all nonlinearity *P* < 0.05). Segmented neutrophil count and alkaline phosphatase showed significant negative effects, with the estimated ALMBMI difference shifting from positive to negative at 4.00 (1000 cells/uL) and 76.00 IU/L, respectively. Creatinine showed a significant positive effect, with the estimated ALMBMI difference shifting from negative to positive at 62.76 umol/L, as shown in Fig. 3.

**Fig. 3:**
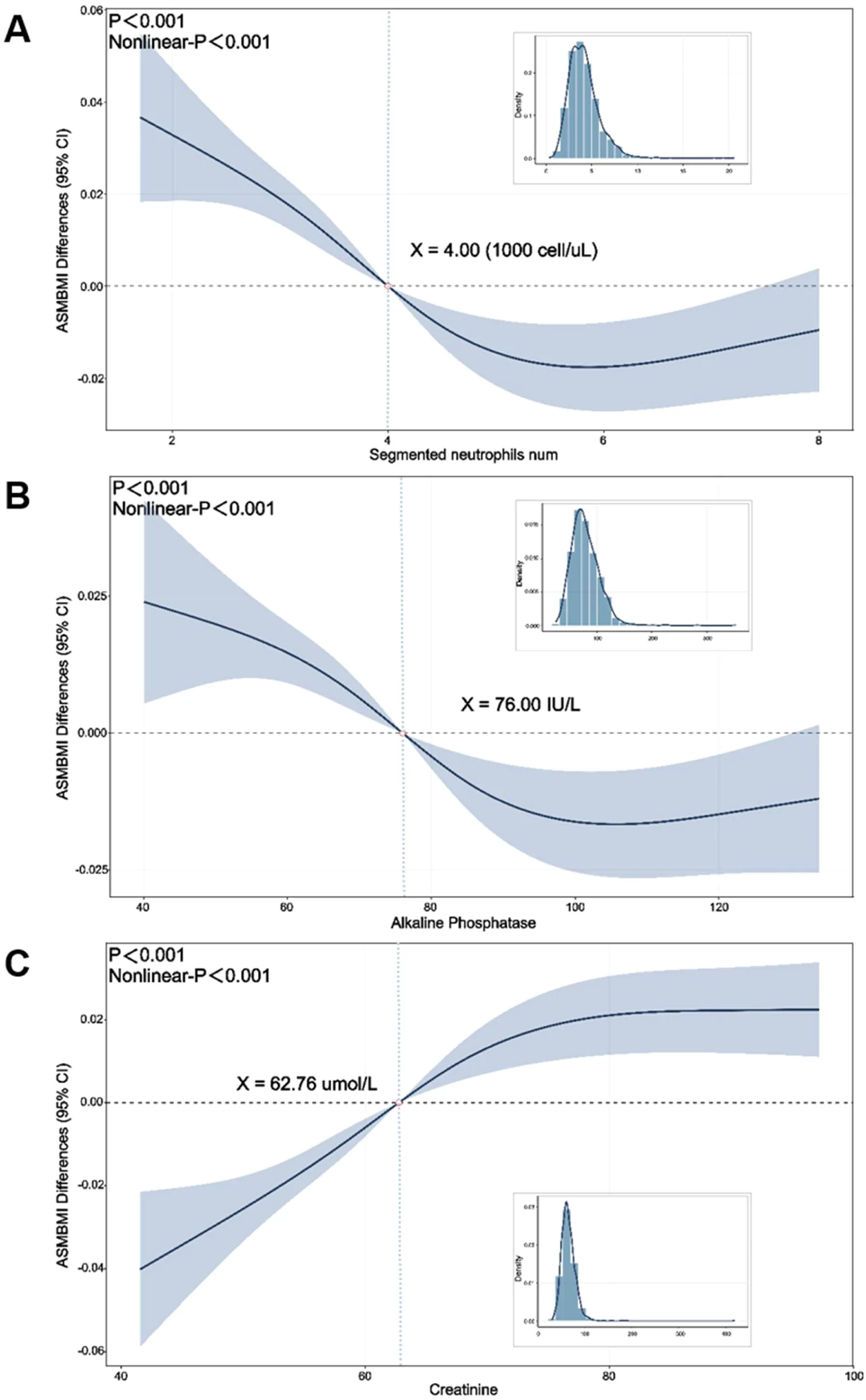
Dose-response relationships between key blood indicators and ALMBMI in the RCS model. **Note:** (A) Dose-response relationship between segmented neutrophil count and ALMBMI. (B) Dose-response relationship between alkaline phosphatase and ALMBMI. (C) Dose-response relationship between creatinine and ALMBMI.

### 3.6 Identifying key blood indicators associated with low ALMBMI

To determine the quantitative relationship between blood variables and low ALMBMI, low ALMBMI status was defined according to the FNIH criteria. A binary logistic regression model was then fitted with the categorical variable as the dependent variable (0 = normal ALMBMI, 1 = low ALMBMI) and the key blood indicators for females as independent variables, with full adjustment for confounders (Table 5). The direction of associations for the different indicators was consistent with the previous analyses. In the female population, creatinine was significantly lower in the low ALMBMI group (*P* < 0.001), while ALP and segmented neutrophil count were significantly higher (*P* < 0.01). Compared with females who had normal ALMBMI, those with low ALMBMI exhibited 16.01% higher ALP and 11.17% higher segmented neutrophil counts. These modest increases in circulating inflammatory markers align with features of chronic inflammation.

**Table 5:** Changes and Comparisons of Key Blood Indicators by ALMBMI status.

Variable	Low ALMBMI	Normal ALMBMI	*P*	*OR*	95% CI
Alkaline phosphatase (IU/L)	90.09±28.31	77.66±26.50	<0.001	1.01	1.01,1.02
Segmented neutrophil count (1000 cells/uL)	4.58±1.54	4.12±1.87	0.005	1.13	1.04,1.23
Creatinine (umol/L)	58.24±19.56	65.99±18.20	<0.001	0.96	0.95,0.98

**Note:** Data are presented as mean ± *SD. OR* were derived from binary logistic regression adjusted for confounders, with normal ALMBMI as the reference group. *CI*, confidence interval; *OR*, odds ratio.

## 4 Discussion

Using a multimodal machine learning framework, this study found that blood indicators from routine health check-ups in middle-aged women can provide valuable information for identifying low ALMBMI. In women with low ALMBMI, elevated segmented neutrophil count and alkaline phosphatase levels were observed, along with decreased creatinine levels. All three core blood indicators exhibited L-shaped or reverse L-shaped nonlinear associations with ALMBMI, with clear threshold effects: below the inflection point, elevated segmented neutrophil count and alkaline phosphatase, as well as decreased creatinine, were associated with significant reduction in ALMBMI; above the inflection point, the associations reached saturation and the curves plateaued. The coordinated changes of these indicators suggest that middle-aged women with low ALMBMI exhibit features consistent with chronic inflammation.

In this study, ALMBMI in women showed a decreasing trend with age, indicating that the loss of appendicular lean mass begins as early as middle age, which is consistent with findings from eight Asian cohorts in Taiwan, Japan, and Malaysia [20]. Throughout human evolution, women's societal roles and division of labor have largely involved tasks requiring endurance and fat storage, such as domestic work and child-rearing, which may accelerate muscle loss[21]. Evidence from human anatomical studies indicates that age-related atrophy of thigh muscles in women begins at age 50, accompanied by significant reductions in muscle mass, as well as marked decreases in muscle fiber number and cross-sectional area [22]. This study observed that individuals who engaged in physical exercise had higher ALMBMI than those who did not, which is largely consistent with existing sarcopenia intervention strategies (e.g., progressive high-intensity resistance training performed on alternating days, 2–4 days per week). Nutritional and exercise interventions during middle age may slow the rate of lean loss in old age and counteract muscle dysfunction as well as neuromuscular damage caused by aging[23^-^24]. Regarding differences in ALMBMI by disease status, individuals with medical conditions had lower ALMBMI than those without, suggesting the existence of interacting molecular pathways between the muscular and skeletal systems, as well as between muscle and other physiological systems[25].

Alkaline phosphatase (ALP) accounted for the highest proportion (36.4%) among factors positively associated with low ALMBMI in middle-aged women and exhibited a nonlinear association with ALMBMI, with an inflection point at 76.00 IU/L. ALP is an important hydrolase whose core component is tissue-nonspecific alkaline phosphatase (TNSALP), primarily produced by bone, liver, and kidney[26]. Clinical studies have indicated that patients with hypophosphatasia carrying TNSALP gene mutations commonly present with symptoms such as muscle dysfunction and musculoskeletal pain. Following seven months of treatment with recombinant TNSALP enzyme, muscle strength significantly improved by 30%[27]. Meanwhile, serum ALP level is also a biomarker of chronic inflammation. Elevated ALP levels not only predict a low-grade inflammatory state in patients with sarcopenia and comorbid liver disease[28] but also reflect inflammatory responses directly associated with malignant tumor metabolism[29]. In our study, after adjusting for various diseases, ALP level consistently demonstrated a stable positive association with low ALMBMI.

Segmented neutrophil count ranked second in weight proportion, accounting for 36.0%, and exhibited an L-shaped nonlinear association with ALMBMI. Segmented neutrophils represent the fully mature form of neutrophils, and their proportion and count are core indicators for assessing infection and inflammation[30]. In the inflammatory microenvironment, neutrophils rapidly infiltrate to clear necrotic cells and debris. However, prolonged presence of neutrophils at the site of injury can impede muscle regeneration. The factors they secrete, such as elastase, while stimulating the proliferation of myogenic progenitor cells, simultaneously inhibit their differentiation and the maturation of muscle fibers[31]. These findings suggest that elevated segmented neutrophil count may contribute to low ALMBMI decline through both inflammatory infiltration and direct interference with myogenic regeneration, supporting its role as a factor associated with low ALMBMI in middle-aged women.

Creatinine accounted for 27.6% of the weight proportion. Although relatively lower than the previous two indicators, it still constituted a core factor and exhibited a reverse L-shaped nonlinear association with ALMBMI. Creatinine is an end product of muscle metabolism, generated by non-enzymatic dehydration of creatine and phosphocreatine, and its serum level is positively correlated with muscle mass[32]. Under normal kidney function, a decreased creatinine level often indicates reduced ALM. The reverse L-shaped association observed in this study further suggests a threshold effect for the association of creatinine with ALMBMI: when creatinine levels fell below 62.76 μmol/L, ALMBMI decreased significantly as creatinine declined; beyond 62.76 μmol/L, the effect reached saturation. Of note, creatinine is also a routine clinical indicator for assessing kidney function, and patients with chronic kidney disease have a significantly higher risk of muscle loss[33^-^34]. In the present study, even after adjusting for kidney-related diseases, the association of creatinine remained stable, suggesting that it reflects muscle metabolic reserve rather than kidney function alone. Therefore, as a factor associated with ALMBMI, creatinine may complement inflammatory indicators to collectively provide a hematological basis for identifying low ALMBMI in middle-aged women.

This study employed a machine learning framework integrating multiple mathematical models, with a focus on the screening, weighting, and directional associations of blood-based indicators. It is the first to elucidate the characteristics and weights of blood-based indicators associated with low ALMBMI in middle-aged women, and to identify the chronic inflammatory profile of individuals with low ALMBMI, thereby providing population-based epidemiological clues for sarcopenia prevention. However, mathematical models serve only as auxiliary tools in research, and the absence of certain indicators from the findings should not be interpreted as evidence of their unimportance. In fact, muscle atrophy and aging involve a complex physiological process in which interactions among multiple indicators collectively shape the molecular pathways underlying muscle wasting. The key indicators identified in this study are of relatively high importance and warrant greater attention from clinicians. Nevertheless, our conclusions are based on observational data and are limited in their ability to establish causal relationships. Prospective studies are needed to elucidate longitudinal associations and the potential influence of temporal factors.

## 5 Conclusions

In summary, we identified core blood indicators associated with low ALMBMI in community-dwelling middle-aged women, all of which are consistent with features of chronic inflammation. These findings support low-cost population-level assessment of inflammatory markers and screening for low appendicular lean mass.

## Ethics statement

This study used publicly available, de-identified data from the National Health and Nutrition Examination Survey (NHANES). The studies involving human participants were reviewed and approved by the NCHS Research Ethics Review Board (ERB) (Protocol #2011–17 and #2018–01, https://www.cdc.gov/nchs/nhanes/irba98.htm). All participants provided written informed consent. As this was a secondary analysis of anonymized data, no additional ethical approval was required. The study was conducted in accordance with the Declaration of Helsinki.

## Declaration of generative AI and AI-assisted technologies in the writing process

During the preparation of this work, the authors used AI-assisted tools solely for language polishing and grammar checking. Following the use of these tools, the authors thoroughly reviewed and edited the content as necessary and take full responsibility for the final version of the manuscript.

## Funding

Key Discipline Projects of Huadong Hospital (LCZX2202; Z.B), and 10.13039/501100012150Shanghai Leading Project of the Oriental Talent Program (No.002; Z.B).

## CRediT authorship contribution statement

**Shanlan Yang:** Writing – review & editing, Writing – original draft, Visualization, Software, Methodology, Formal analysis, Data curation, Conceptualization. **Jiaofeng Wang:** Writing – review & editing, Resources, Funding acquisition. **Dayoung Kim:** Writing – review & editing. **Han Xu:** Writing – review & editing. **Sheng Zhou:** Writing – review & editing. **Fan Yang:** Writing – review & editing, Writing – original draft, Validation, Resources, Methodology, Funding acquisition, Conceptualization. **Zhijun Bao:** Writing – review & editing, Validation, Supervision, Funding acquisition, Conceptualization.

## Declaration of competing interest

The authors declare that they have no known competing financial interests or personal relationships that could have appeared to influence the work reported in this paper.

## Data Availability

The datasets presented in this study can be found in online repositories. The names of the repository/repositories and accession number(s) can be found below: This study examined datasets that were accessible to the public. The location of this data can be accessed here: http://www.cdc.gov/nhanes.
