# Supplementary material for: Association of alkaline phosphatase and segmented neutrophils count with low appendicular lean mass in middle-aged women: Evidence from an American cross-sectional survey
Source: J Frailty Aging. 2026 Oct 13;15(6):100213. doi: 10.1016/j.tjfa.2026.100213 (PMC13685937; doi:10.1016/j.tjfa.2026.100213)
Supplement: Supplementary file 1 [file mmc1.docx]

**Supplementary Files Checklist**

| **Page 1** | **Supplementary Files Checklist** |
| --- | --- |
| **Page 2** | **Supplementary Figure1 Correlation plots of variables in female** |
| **Page 3** | **Supplementary Figure2 Hyperparameter optimization curves for elastic linear logistic regression in female.** |
| **Page 4** | **Supplementary Figure3 Trajectory plots for global and three exposure variables in female** |
| **Page 5** | **Supplementary Table 1 Serial correlation lag coefficients for female exposure variables in the BKMR model** |

**Supplementary Figures**

**Supplementary Figure1 Correlation plots of variables in female**

**Supplementary Figure2 Hyperparameter optimization curves for elastic linear logistic regression in female.**

**Supplementary Figure3 Trajectory plots for global and three exposure variables in female**

**Supplementary Tables**

| **Supplementary Table 1 Serial correlation lag coefficients for female exposure variables in the BKMR model** | | | | |
| --- | --- | --- | --- | --- |
| **Lag** | **lambda** | **r1** | **r2** | **r3** |
| 0 | 1.000 | 1.000 | 1.000 | 1.000 |
| 1 | 0.800 | 0.961 | 0.934 | 0.928 |
| 2 | 0.651 | 0.922 | 0.864 | 0.868 |
| 3 | 0.536 | 0.879 | 0.788 | 0.812 |
| 4 | 0.446 | 0.835 | 0.710 | 0.751 |
| 5 | 0.372 | 0.791 | 0.651 | 0.694 |
| 6 | 0.312 | 0.747 | 0.594 | 0.634 |
| 7 | 0.260 | 0.702 | 0.545 | 0.579 |
| 8 | 0.221 | 0.668 | 0.494 | 0.536 |
| 9 | 0.184 | 0.632 | 0.440 | 0.490 |
| 10 | 0.155 | 0.596 | 0.385 | 0.442 |
| 11 | 0.129 | 0.558 | 0.327 | 0.397 |
| 12 | 0.109 | 0.520 | 0.268 | 0.359 |
| 13 | 0.086 | 0.480 | 0.225 | 0.317 |
| 14 | 0.070 | 0.439 | 0.179 | 0.274 |
| 15 | 0.053 | 0.395 | 0.140 | 0.228 |
| 16 | 0.039 | 0.349 | 0.100 | 0.191 |
| 17 | 0.030 | 0.303 | 0.064 | 0.163 |
| 18 | 0.025 | 0.262 | 0.043 | 0.133 |
| 19 | 0.023 | 0.222 | 0.040 | 0.109 |
| 20 | 0.022 | 0.191 | 0.039 | 0.096 |
| 21 | 0.020 | 0.159 | 0.037 | 0.086 |
| 22 | 0.020 | 0.128 | 0.035 | 0.077 |
| 23 | 0.016 | 0.109 | 0.033 | 0.067 |
| 24 | 0.013 | 0.089 | 0.032 | 0.060 |
| 25 | 0.010 | 0.069 | 0.030 | 0.052 |
| 26 | 0.004 | 0.055 | 0.028 | 0.045 |
| 27 | 0.003 | 0.040 | 0.026 | 0.037 |
| 28 | 0.000 | 0.031 | 0.023 | 0.029 |
| 29 | -0.002 | 0.021 | 0.020 | 0.020 |
| 30 | -0.006 | 0.011 | 0.018 | 0.013 |
